# Synthesis and Characterization of CuFe_2_O_4_ Nanoparticles Modified with Polythiophene: Applications to Mercuric Ions Removal

**DOI:** 10.3390/nano10030586

**Published:** 2020-03-23

**Authors:** Ayman H. Kamel, Amr A. Hassan, Abd El-Galil E. Amr, Hadeel H. El-Shalakany, Mohamed A. Al-Omar

**Affiliations:** 1 Chemistry Department, Faculty of Science, Ain Shams University, Abbasia 11566, Egypt; amr_hassan@sci.asu.edu.eg (A.A.H.); hadeelhesham100@gmail.com (H.H.E.-S.); 2 Department of Chemistry, Virginia Commonwealth University, Richmond, VA 23284, USA; 3 Pharmaceutical Chemistry Department, Drug Exploration & Development Chair (DEDC), College of Pharmacy, King Saud University, Riyadh 11451, Saudi Arabia; malomar1@ksu.edu.sa; 4 Applied Organic Chemistry Department, National Research Center, Dokki 12622, Egypt

**Keywords:** CuFe_2_O_4_ nanoparticles, CuFe_2_O_4_@Polythiophene composite, mercury (II) removal, adsorption

## Abstract

In this research, CuFe_2_O_4_ nanoparticles were synthesized by co-precipitation methods and modified by coating with thiophene for removal of Hg(II) ions from aqueous solution. CuFe_2_O_4_ nanoparticles, with and without thiophene, were characterized by x-ray diffraction (XRD), Field emission scanning electron microscopy (FESEM), energy dispersive x-ray (EDX), high-resolution transmission electron microscopy (HRTEM) and Brunauer–Emmett–Teller (BET). Contact time, adsorbent dose, solution pH, adsorption kinetics, adsorption isotherm and recyclability were studied. The maximum adsorption capacity towards Hg^2+^ ions was 7.53 and 208.77 mg/g for CuFe_2_O_4_ and CuFe_2_O_4_@Polythiophene composite, respectively. Modification of CuFe_2_O_4_ nanoparticles with thiophene revealed an enhanced adsorption towards Hg^2+^ removal more than CuFe_2_O_4_ nanoparticles. The promising adsorption performance of Hg^2+^ ions by CuFe_2_O_4_@Polythiophene composite generates from soft acid–soft base strong interaction between sulfur group of thiophene and Hg(II) ions. Furthermore, CuFe_2_O_4_@Polythiophene composite has both high stability and reusability due to its removal efficiency, has no significant decrease after five adsorption–desorption cycles and can be easily removed from aqueous solution by external magnetic field after adsorption experiments took place. Therefore, CuFe_2_O_4_@Polythiophene composite is applicable for removal Hg(II) ions from aqueous solution and may be suitable for removal other heavy metals.

## 1. Introduction

Heavy metal pollution in aqueous solution is a critical environmental problem [1,2]. Mercury is one of the most toxic pollutants for human and animals, even at low levels [3]. It is produced naturally from various natural sources such as volcanic eruption, weathering of rocks and soils [4]. It is also produced from different industrial sources such as pharmaceuticals, chloralkali, plastic, textile, paint, rubber, paper, cement, electronic industry, coal combustion, fertilizers, oil refining and rubber processing [5,6]. It exists in various forms such as metallic mercury, mercurous (Hg^+^), mercuric (Hg^2+^) and organic mercury containing phenyl, methyl and ethyl groups, etc. It causes various diseases such as Alzheimer’s disease, amyotrophic lateral sclerosis, Parkinson’s disease and damaging of the immune system and kidneys. Hence, mercury is considered as prior hazardous pollutant by the Agency for Toxic Substances and Disease Registry [7]. According to World Health Organization (WHO), 1 µg/L is the maximum permissible concentration of Hg(II)in drinking water [8]. According to the European Union (EU), the maximum acceptable level of Hg(II) is 5 µg/L for wastewater discharge [9,10,11].

Various physical and chemical methods have been used for elimination of mercury from polluted water including adsorption, ion exchange, coagulation, membrane filtration, electrochemical treatment, solvent extraction and chemical precipitation [9,10,11,12,13,14,15,16,17,18]. However, adsorption techniques are distinguished by simple and low-cost techniques and reusability of adsorbent [19,20,21,22].

Nowadays, there is a focus on the application of nanomaterials in the removal of environmental pollutants. This is based on their distinctive properties such as high surface area, high adsorption and special photoelectric property. However, they are suffering from difficulty of their separation from aqueous solutions due to their small particle size which restricts the application in water treatment. So, it is preferable using magnetic nanomaterials that can be easily separated from solution with external magnetic field [23,24,25].

According to “Pearson’s hard soft acid-base theory (HSAB)”, mercury is classified as a soft acid that forms strong bonds with soft base groups such as –CN, –RS and SH [26]. As such, if the adsorbent contains soft base groups, it can easily eliminate mercury from the solution.

In this study, CuFe_2_O_4_ was modified by thiophene polymer to improve its adsorption property of mercury from solution. CuFe2O_4_ was synthesized and characterized before and after polymerization. Their adsorption and desorption behaviors towards Hg^2+^ ions were studied.

## 2. Experimental

### 2.1. Reagents and Instruments

All reagents are used without further purification. Ferric sulfate hydrate, copper sulfate pentahydrate and ammonium persulfate were purchased from Sigma–Aldrich (St. Louis, MO, USA). Thiophene was acquired from Fluka (Ronkonoma, NY, USA). Cetyltrimethylammonium bromide (CITAB) was obtained from BDH (Darmstadt, Germany). Atomic absorption spectrometer (Thermo scientific S4 series, AA Spectrometer with continuous flow vapor (VP100), with auto sampler (CETAC 520), background correction, deuterium lamp, hollow-cathode lamps (HCL) specific for each element Hg and a computer with software of SOLLAR AAS SYSTEM) was used to determine the concentration of mercuric ions (Illinois, IL, USA). The prepared adsorbents were characterized by x-ray diffraction (XRD) which carried out by X’Pert-PRO-PANalytical x-ray diffractometer (PANalytical, Almelo, the Netherlands) using CuKα radiation (*λ* = 1.5406Å) in the 2*θ* range from 10 °C to 80 °C High-resolution transmission electron microscopy (HRTEM) images were taken by JEOL-JEM-2100 electron microscope instrument (Osaka, Japan). Field emission scanning electron microscopy (FESEM) images were taken by QuantaFEG250 instrument (Kolkata, India) equipped with an energy dispersive x-ray analysis (EDX) to study the surface morphology and the chemical composition of adsorbents before and after adsorption process. Brunauer–Emmett–Teller (BET) method was performed by adsorption–desorption of N_2_ gas at 77 °C using Nova 3200 s unit instrument (Florida, FL, USA) to determine the surface area and pore size distribution of adsorbents.

### 2.2. Adsorbents Preparation

#### 2.2.1. Synthesis of CuFe_2_O_4_

Copper ferrite nanoparticles were synthesized using a co-precipitation technique. Typically, 0.02 mol Fe_2_(SO_4_)_3_ and 0.01mol CuSO_4_ were dissolved in 1 L of 0.01 mol/L Cetyltrimethylammonium bromide (CTAB) solution, as a capping agent. The mixture was kept under stirring for an hour to ensure reaching the equilibrium. The pH of the solution was then adjusted to pH 10 using NaOH solution. The mixture was kept under stirring for two extra hours and then aged overnight. The obtained precipitate was washed several times with water until a neutral filtrate was obtained and then dried at 60 °C overnight. The obtained brown black product was then calcined at 500 °C for 4 h. The obtained nano ferrite was then ground in a porcelain mortar and the powder was kept in a desiccator for further characterization and experiments.

#### 2.2.2. Synthesis of CuFe_2_O_4_@Polythiophene Composite

The ferrite polymeric composite was synthesized according to the following: 4.5 g of CuFe_2_O_4_ and 20 mL chloroform were added into 30 mL distilled water then the resulting mixture was dispersed by an ultrasonic bath at room temperature for about 1h. Six grams of thiophene monomer was added slowly into the mixture. Three grams of ammonium persulfate (APS) was dissolved in 20 mL of distilled water then dropped into the mixture within 30 min under sonication. The resulting mixture was left for 24 h at room temperature to allow the reaction takes place then poured the reaction mixture into acetone. The obtained precipitate was filtered and washed several times by distilled water then by methanol. The obtained precipitate was dried at 50 °C then thoroughly ground it to obtain fine particles.

### 2.3. Adsorption Techniques

The batch equilibrium method was carried out to study the adsorption capacities and removal efficiency of adsorbents after equilibrium took place. Three-tenths of a gram of adsorbents was dispersed in 50 mL of aqueous Hg^2+^ solutions of different concentrations and stirred 3 h for CuFe_2_O_4_ and 30 min. for CuFe_2_O_4_@Polythiophene composite at room temperature then filtered the adsorbent by 0.22µm Millipore then the concentration of Hg^2+^ ions in filtrate was measured by atomic absorption. Eventually, the adsorption capacities (mg/g) after equilibrium and removal efficiency of both adsorbents were calculated by Equations (1) and (2) [27]:

*Q_e_* = (*C_o_* − *C_e_*) × *V*/*M* (1)

(2) $$\mathrm{Removal}\;\mathrm{efficiency}\;\mathrm{of}\;\mathrm{Hg}2^{+}\;\mathrm{ions}\;\%=\frac{\left(Co-Ce\right)}{Co}\;\times \;100$$

where; *C_o_* is the initial concentration of Hg^2+^ ions (mg/L), *C_e_* is the concentration of Hg^2+^ ions at equilibrium (mg/L), *V* is the volume of initial aqueous solution of Hg^2+^ ions (L) and *M* is the mass of adsorbent (g).

### 2.4. Adsorption Kinetics

The kinetic studies of Hg(II) ions adsorption on CuFe_2_O_4_ nanoparticles and CuFe_2_O_4_@Polythiophene composite were investigated by exposure 30 mg of both adsorbents to 50 mL of 10 mg/L Hg(II) ion solutions of pH 6 at different times. For analyzing the adsorption rate, pseudo first order and second order models as shown in Equations (3) and (4) [27]; respectively, were utilized to appropriate the practical.

ln(*q_e_* – *q_t_*) = ln *q_e_* – *k_1_t* (3)

*t/q_t_* = (1/*k_2_q_e_*^2^) + (*t/q_e_*) (4)

where; *q_e_* (mg/g) and *q_t_* (mg/g) are the adsorption capacity of adsorbents at equilibrium and at any time; respectively, *t* (min) is time, *k_1_* (min^-1^) and *k_2_* (g·mg^−1^·min^−1^) are the rate constants of pseudo first order and second order adsorption; respectively.

### 2.5. Impact of Hg(II) Solution pH

The influence of solution pH on Hg(II) ions adsorption for both adsorbents was examined by contact 30 mg of adsorbent to 50 mL of 10 mg/L Hg(II) solutions in the pH range 2–7. The pH of Hg(II) solutions was adjusted by using HNO_3_ and NaOH.

### 2.6. Adsorption Isotherms

The adsorption isotherms of both adsorbents were performed by adding 30 mg of adsorbents to 50 mL Hg(II) ion solutions of different concentrations (3.5–209.3 mg/L) at pH 7. The contact time using CuFe_2_O_4_ and CuFe_2_O_4_@Polythiophene composite was 3 h and 30 min, respectively. The mixture was filtered then the concentration of the remained Hg^2+^ ions in the filtrate was measured using atomic absorption spectrometry (AAS).

The experimental data obtained are contrasted using Langmuir and Freundlich adsorption models [27,28]. The Langmuir and Freundlich models can be expressed as shown in Equations (5) and (6); respectively:

(*C_e_/q_e_*) = (1/*a_L_q_m_*) + (*C_e_/q_m_*) (5)

log *q_e_* = log *k_F_* + (1*/n*) log *C_e_* (6)

where; *C_e_* (mg/L) is the concentration of Hg(II) solution at equilibrium, *q_e_* (mg/g) is the equilibrium adsorption capacity of sorbent, *q_m_* (mg/g) is the maximum adsorption capacity of adsorbent, *a_L_* is the Langmuir constant, *k_F_* is the Freundlich constant and (*1/n*) is the heterogeneity factor. The adsorption process becomes favorable and the surface of adsorbent is heterogeneous when the value of *n* is located between 1 and 10 [24].

### 2.7. Regeneration of CuFe_2_O_4_@Polythiophene Composite

The reusability of CuFe_2_O_4_@Polythiophene composite was tested by performing five alternating adsorption–desorption cycles. Each adsorption cycle was performed as mentioned previously then the adsorbent was extracted from solution by using external magnetic field. The extracted adsorbent was dispersed for 30 min in 20 mL of 0.5 mol/LHCl then washed several times by distilled water till the pH of filtrate becomes 6. The re-generated adsorbent was used for sequential adsorption–desorption cycles and the Hg(II) adsorption efficiency was obtained by using Equation (2).

## 3. Results and Discussions

### 3.1. Characterization of CuFe_2_O_4_ and CuFe_2_O_4_@Polythiophene Composite

#### 3.1.1. X-Ray diffraction (XRD) Analysis

**Figure 1:**
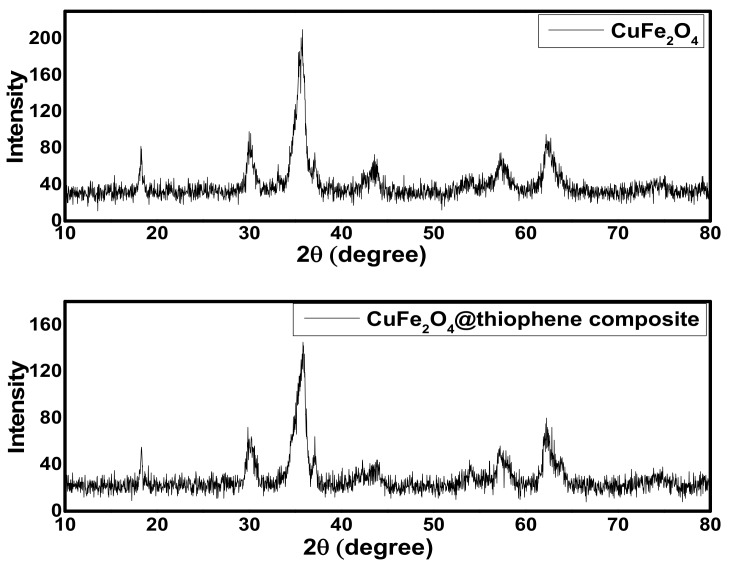
The x-ray diffraction (XRD) patterns of CuFe_2_O_4_ nanoparticles and CuFe_2_O_4_@Polythiophene composite.

As shown in Figure 1, the XRD patterns of both CuFe_2_O_4_ and CuFe_2_O_4_@thiophene composite indicated that the main phase of both samples was cuprospinel. The diffraction pattern of CuFe_2_O_4_@Polythiophene composite showed six sharp peaks at 19.1°, 30.8°, 36.4°, 44.2°, 58.7° and 62.8° corresponding to (80), (100), (201), (50), (55) and (88) of CuFe_2_O_4_.The crystalline size of CuFe_2_O_4_ and CuFe_2_O_4_@Polythiophene composite was calculated by Scherer equation (i.e., Equation (7)) [22]. It was 9.1 and 21.1 nm for CuFe_2_O_4_ and CuFe_2_O_4_@Polythiophene composite, respectively.

(7) $$D=\frac{k\lambda }{\beta cos\theta }$$

where; *D* is the crystalline size of sorbent, *λ* is the x-ray’s wavelength which equals 1.54 Å, *β* is the full width at half maximum of the peak (FWHM), *θ* is the diffraction angle (in radians) and k = 0.9.

#### 3.1.2. Field Emission Scanning Electron Microscopy Analysis (FESEM) before and after Adsorption

**Figure 2:**
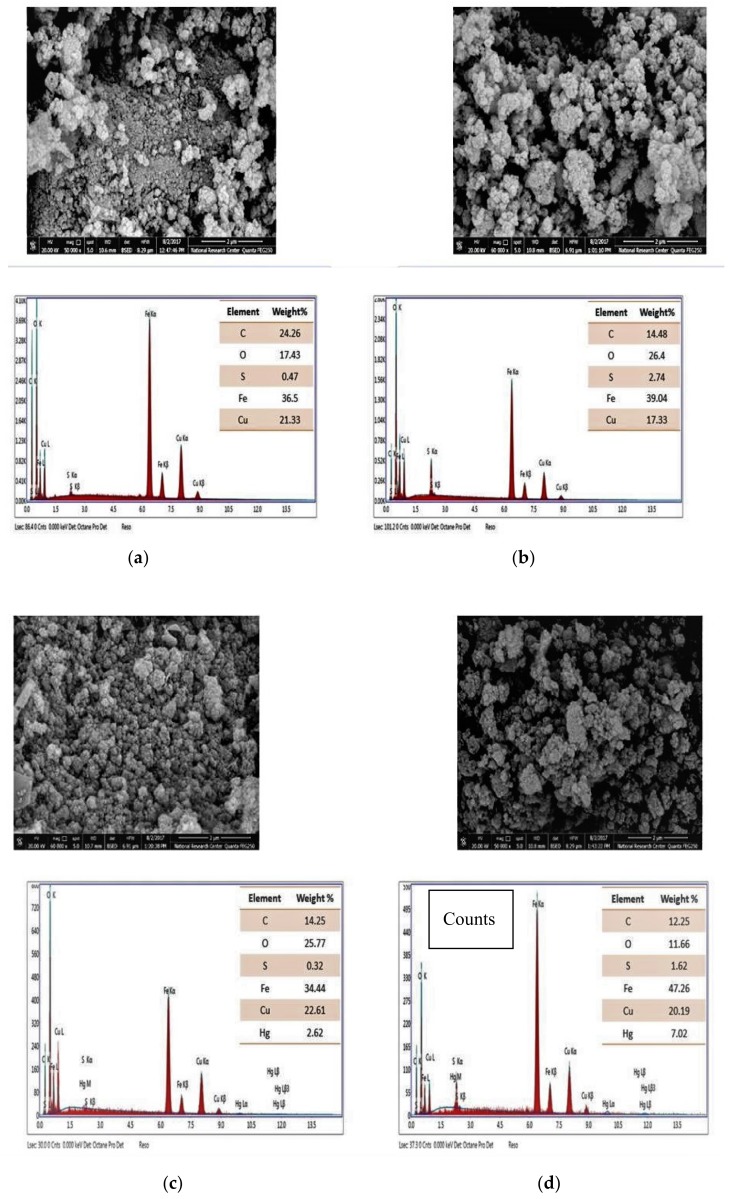
Field emission scanning electron microscopy (FESEM) before adsorption for CuFe_2_O_4_ (**a**) and (**b**) CuFe_2_O_4_@Polythiophene composite) and after Hg^2+^ adsorption (CuFe_2_O_4_ (**c**) and (**d**) CuFe_2_O_4_@Polythiophene composite).

The FESEM images of CuFe_2_O_4_ and CuFe_2_O_4_@Polythiophene composite before and after adsorption process is shown in Figure 2. It indicates that CuFe_2_O_4_ have particle sizes varying from 11.5 to 30 nm, which is in agreement with the crystallite size obtained from XRD data.

The EDX analysis was carried out to clarify the elemental composition of CuFe_2_O_4_ and CuFe_2_O_4_@Polythiophene composite before and after adsorption process as illustrated in Figure 2. EDX spectrum of CuFe_2_O_4_ was clarified that stoichiometric ratio of Cu/Fe =1/2.The sulfur present in CuFe_2_O_4_ may be due to a residue from the starting sulphate salts. It was also found that the percentage of sulfur was increased in CuFe_2_O_4_@Polythiophene composite compare to ferrite alone that revealed that the polymerization of thiophene in the surface of CuFe_2_O_4_ took place. The two prepared materials showed a peak corresponding to mercury with a higher percentage in the polymer composite compared to the ferrite alone. This indicate the favorable adsorption Hg(II) ions were on CuFe_2_O_4_@Polythiophene. The spectrums were free from any other metal ions which reveal the purity of the prepared samples.

#### 3.1.3. High-Resolution Transmission Electron Microscopy (HRTEM)

**Figure 3:**
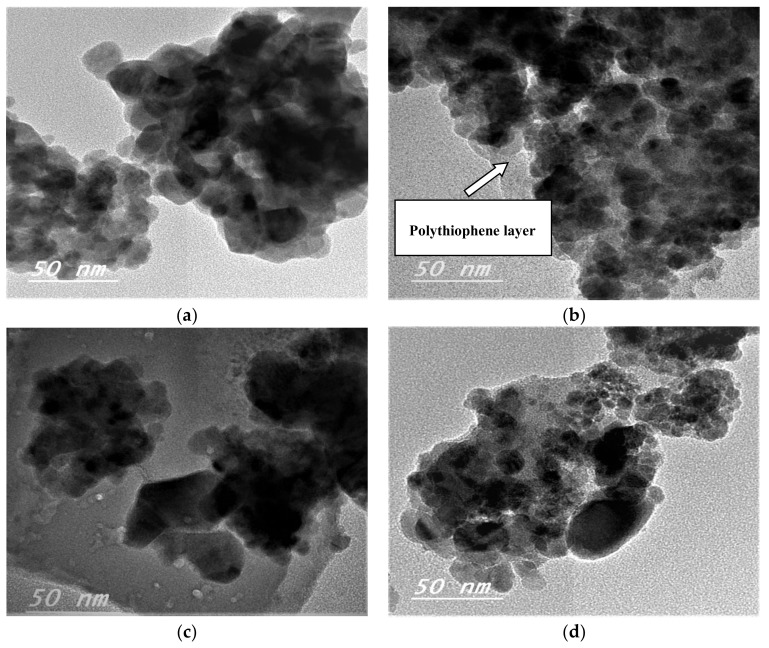
High-resolution transmission electron microscopy (HRTEM) images before adsorption for [A] (CuFe_2_O_4_ (**a**) and (**b**) CuFe_2_O_4_@Polythiophene composite) and after Hg^2+^ adsorption [B] (CuFe_2_O_4_ (**c**) and (**d**) CuFe_2_O_4_@Polythiophene composite).

The morphology and average particle size of the prepared CuFe_2_O_4_ and CuFe_2_O_4_@Polythiophene composite nanocomposites were examined by high-resolution transmission electron microscopy (HR-TEM). As shown in Figure 3, it illustrates the HRTEM images of CuFe_2_O_4_ and CuFe_2_O_4_@Polythiophene composite before and after adsorption process. The images clarified that the particles were in nano size range with spherical shape which are in agreement with XRD and FESEM results. The HRTEM images of CuFe_2_O_4_@Polythiophene composite illustrate that appearance of thiophene polymer layer on the surface of CuFe_2_O_4_ nanoparticles.

#### 3.1.4. Surface Area and Pore Structure

**Figure 4:**
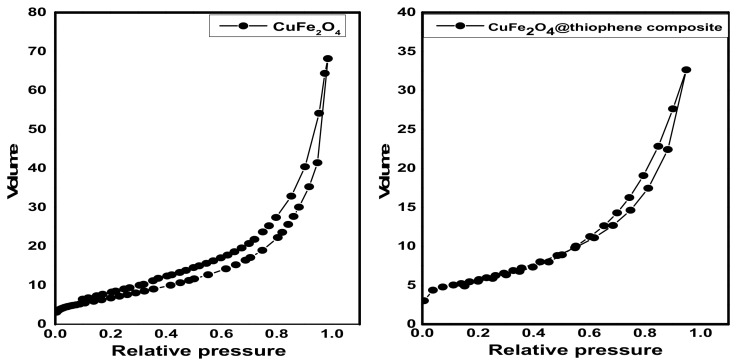
N_2_ adsorption–desorption isotherms of CuFe_2_O_4_ and CuFe_2_O_4_@Polythiophene composite.

The surface area, pore size and pore diameter are estimated by Brunauer–Emmett–Teller (BET) method and illustrated in Table 1. As shown in Figure 4, it represents the N_2_adsorption–desorption isotherms at 77 °C on fabricated adsorbents. The adsorption isotherms of CuFe_2_O_4_ and CuFe_2_O_4_@Polythiophene composite are classified as type II with H_3_ hysteresis loop according to IUPAC which indicates un-restricted monolayer-multilayer adsorption and the sample was plate-like particles with slit-shaped pores.

**Figure 5:**
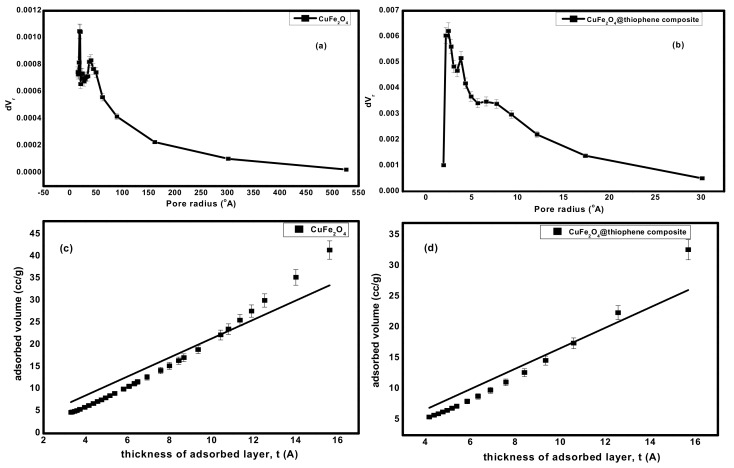
(**a**,**b**) pore size distribution of CuFe_2_O_4_ and CuFe_2_O_4_@Polythiophene composite; respectively and (**c**,**d**) t-plot of CuFe_2_O_4_ and CuFe_2_O_4_@Polythiophene composite; respectively.

BET surface area of CuFe_2_O_4_@Polythiophene composite was significantly higher than CuFe_2_O_4_ that provide more adsorption sites for Hg(II). As illustrated in Figure 5, the pore size distribution curve of CuFe_2_O_4_ sample indicates the existence of mixture of micro and mesopores and a little amount of macropores on the other handthe pore size distribution curve of CuFe_2_O_4_@Polythiophene composite sample indicates the majority existence of microspores and also pore size detection was carried out by constructing *V_a_-t* plot. The *V_a_-t* plot of CuFe_2_O_4_ sample illustrates upward and downward deviation that clarified the presence of mixture of micro- and mesopores in the sample on the other hand The *V_a_-t* plot of CuFe_2_O_4_@Polythiophene composite sample illustrates downward and small upward deviation that clarified the majority existence of micropores that is in agreement with pore size distribution curve. The capillary condensation phenomena which appeared in both isotherms as shown in Figure 4, was attributed to the presence of micropores and narrow mesopores.

### 3.2. Adsorption Studies

#### 3.2.1. Adsorption Kinetics

**Figure 6:**
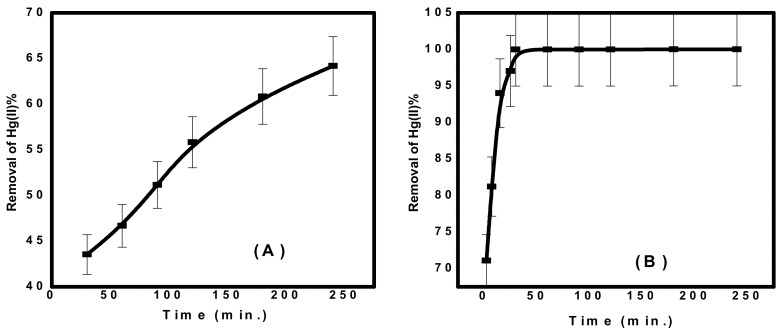
Effect of contact time on Hg(II) removal from aqueous solution (**A**) CuFe_2_O_4_ and (**B**) CuFe_2_O_4_@Polythiophene composite [Conditions: Initial conc. of Hg(II) solution= 10 mg/L, adsorbent dose = 0.05 g, pH = 6, volume of Hg(II) solution = 50 mL).

**Figure 7:**
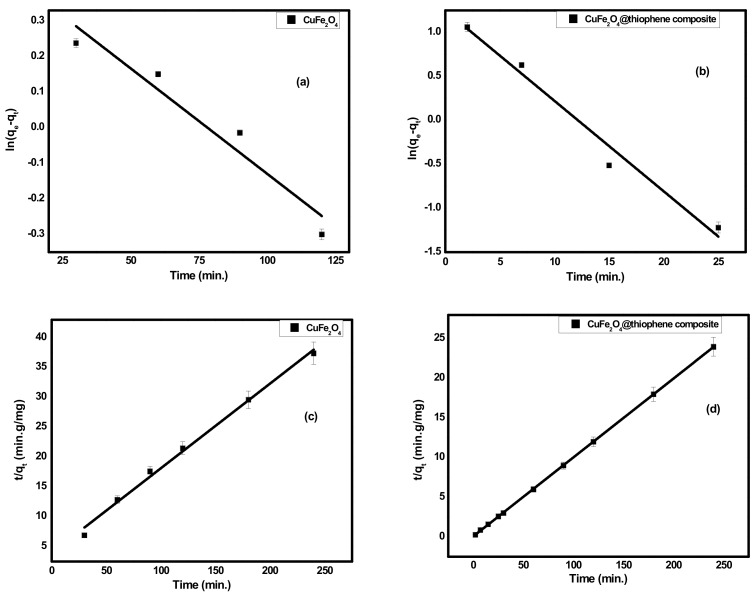
(**a**,**b**) kinetics plot of pseudo first order for adsorption of Hg(II) ions onto CuFe_2_O_4_ and CuFe_2_O_4_@Polythiophene composite; respectively and (**c**,**d**) kinetics plot of second order for adsorption of Hg(II) ions onto CuFe_2_O_4_ and CuFe_2_O_4_@Polythiophene composite, respectively.

As shown in Figure 6, the concentration of Hg(II) ions was studied relative to the contact time of each adsorbent. It was found that the time required to obtain more than 60% of Hg(II) removal was 3 h for CuFe_2_O_4_. However, in case of CuFe_2_O_4_@Polythiophene composite, the time required to achieve the equilibrium was half hour with a removal percentage of 99.98%. Kinetics is the vital feature to investigate the mechanism of adsorption process. The practical kinetics data were fitted with pseudo first order and second order models as shown in Figure 7. The obtained results were illustrated in Table 2. It was observed that the current adsorption process of both adsorbent was obeyed second order model because correlation coefficient (*R^2^*) of second order was higher than pseudo first order and the approximate values between practical and calculated data (*q_e2_* and *q_e_^exp^*) for both adsorbents.

#### 3.2.2. Effect of Adsorbent Dose

**Figure 8:**
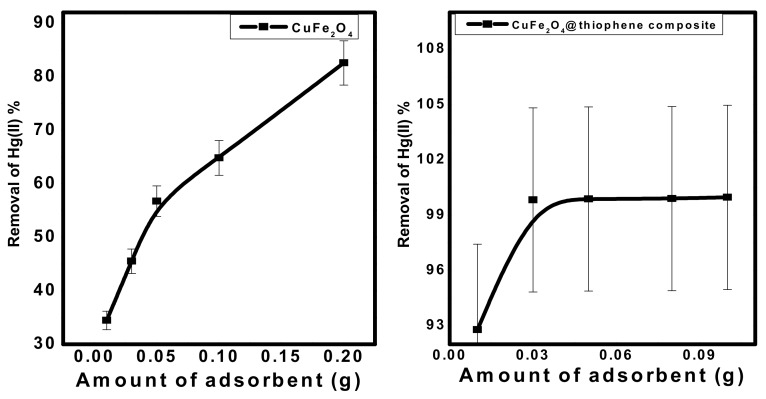
Effect of adsorbent dose on Hg(II) removal from aqueous solution. [Conditions: initial conc. of Hg(II) solution = 10 mg/L, contact time = 3 h and 30 min for CuFe_2_O_4_ and CuFe_2_O_4_@Polythiophene composite; respectively, pH = 6, volume of Hg(II) solution = 50 mL].

Different quantities of the prepared nanomaterials (0.01 to 0.2 g) were mixed with 50 mL (10 mg/L) Hg^2+^ solution at pH 6.0 and stirred. Figure 8 shows that the removal of Hg^2+^ increased from 35% and 92%, with 0.01 g adsorbent to 84.2% and 99.6% for 0.15 g of adsorbent using CuFe_2_O_4_ and CuFe_2_O_4_@Polythiophene composite, respectively. The optimum adsorbent dose was chosen to be 0.03 g for both sorbents.

#### 3.2.3. Effect of pH

**Figure 9:**
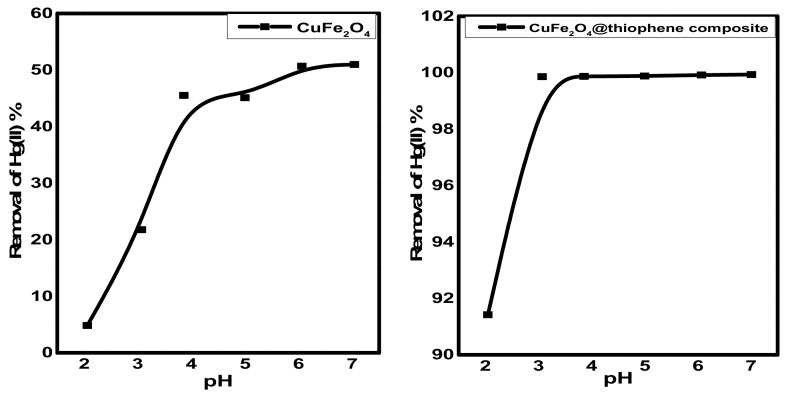
Effect of pH on Hg(II) removal from aqueous solution [Conditions: initial conc. of Hg(II) solution = 10 mg/L, contact time = 3 h and 30 min. for CuFe_2_O_4_ and CuFe_2_O_4_@Polythiophene composite; respectively, adsorbent dose = 0.03 g, volume of Hg(II) solution = 50 mL].

The pH is an important factor for Hg^2+^ adsorption due to its relevance to Hg speciation, as well as the interactions between Hg species and adsorbent surfaces. However, the CuFe_2_O_4_/polythiophene particles prepared in this study exhibited negligible dependence on feed-water pH. When the feed water pH was varied from 3.0 to 7.0, Hg^2+^ removal efficiency of CuFe_2_O_4_/polythiophene particles remained at ~99% (Figure 9). At pH > 7, the Hg(II) ions convert to hydroxide species. For CuFe_2_O_4_particles the removal percentage of Hg(II) ions became constant in pH range of 6–7 for CuFe_2_O_4_. So that pH 6 was chosen for both sorbents.

**Figure 10:**
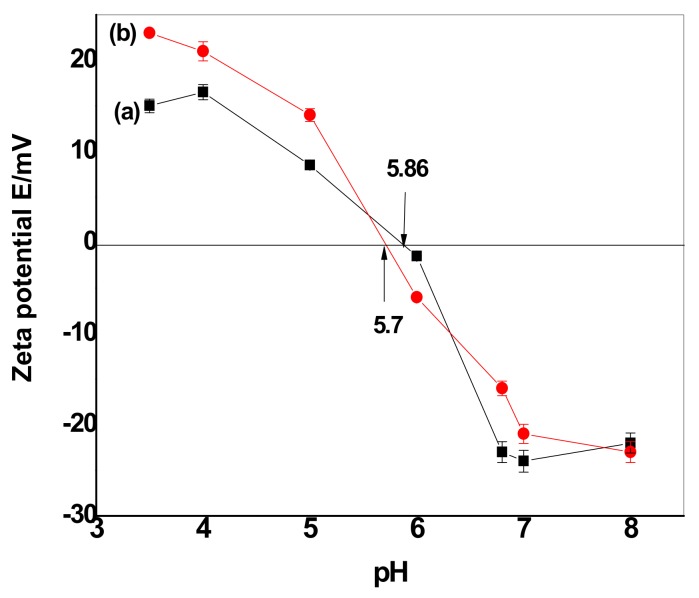
Plots of the zeta potential as a function of pH for (**a**) CuFe_2_O_4_ and (**b**) CuFe_2_O_4_@Polythiophene.

Based upon the zeta potential results (Figure 10), CuFe_2_O_4_ and CuFe_2_O_4_@Polythiophene particles prepared in this study had net negative charges at pH = 6.0 and 8.0 and positive charges at pH = 5.0. The surface charge properties of both CuFe_2_O_4_ and CuFe_2_O_4_@Polythiophene particles were studied by measuring zeta potentials of their aqueous dispersion at varied pH values. As shown in Figure 10, CuFe_2_O_4_ and CuFe_2_O_4_@Polythiophene particles had positive zeta potentials in the pH range of 3.5–5.0. When pH increased from 6.0 to 8, their zeta potentials decreased from 0 to −23 mV, suggesting that the CuFe_2_O_4_ particles became negatively charged at pH=6.0 be attributed to that at low pH, the oxygen-containing groups on the CuFe_2_O_4_ surface will form aqua complexes (i.e., M–OH_2_^+^) and give rise to the formation of positive charge, whereas at higher pH values, oxygen-containing groups (e.g., –OH) are ionized to –O–, forming negative charges on the CuFe_2_O_4_ surface.

#### 3.2.4. Effect of Initial Concentration of Hg(II) Solutions

**Figure 11:**
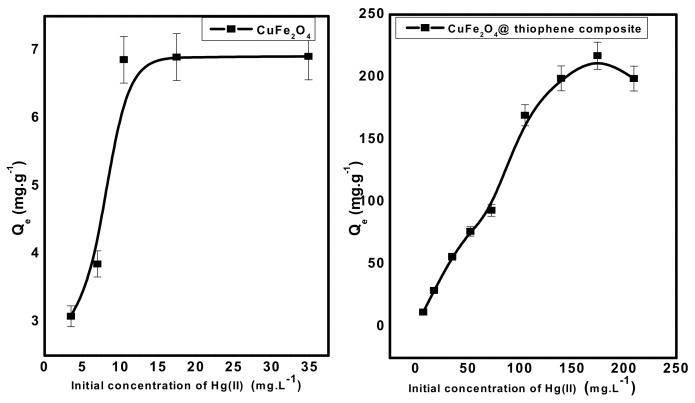
Effect of initial concentration of Hg(II) solution on removal of Hg(II) from aqueous solution. [Conditions: adsorbent dose = 0.03 g, pH = 6, volume of Hg(II) solution = 50 mL, contact time = 3 h and 30 min for CuFe_2_O_4_ and CuFe_2_O_4_@Polythiophene composite; respectively).

The effect of Hg(II) concentration at pH 6 was investigated under the optimized experimental conditions for both adsorbent types. Fifty-milliliter aliquots of Hg^2+^ solution at pH 6.0 was allowed to interact with 0.03 gm adsorbent dose for 30 min. As shown in Figure 11, the adsorption capacity (*Q_e_*) of both adsorbents increased gradually with increasing the concentration of Hg(II) until all active sites of each adsorbent were occupied and there weren’t any more active sites available to occupy. The maximum capacity per unit mass (*Q_m_*) of each adsorbent was 7 and 217 mg/g for CuFe_2_O_4_and CuFe_2_O_4_@Polythiophene composite, respectively. This confirms that CuFe_2_O_4_@Polythiophene composite is more favorable than CuFe_2_O_4_ for Hg(II) removal from aqueous solution. The promising adsorption performance of Hg(II) ions by CuFe_2_O_4_@Polythiophene composite arises from the soft acid–soft base strong interaction between sulfur group of thiophene and Hg(II) ions.

#### 3.2.5. Adsorption Isotherms

**Figure 12:**
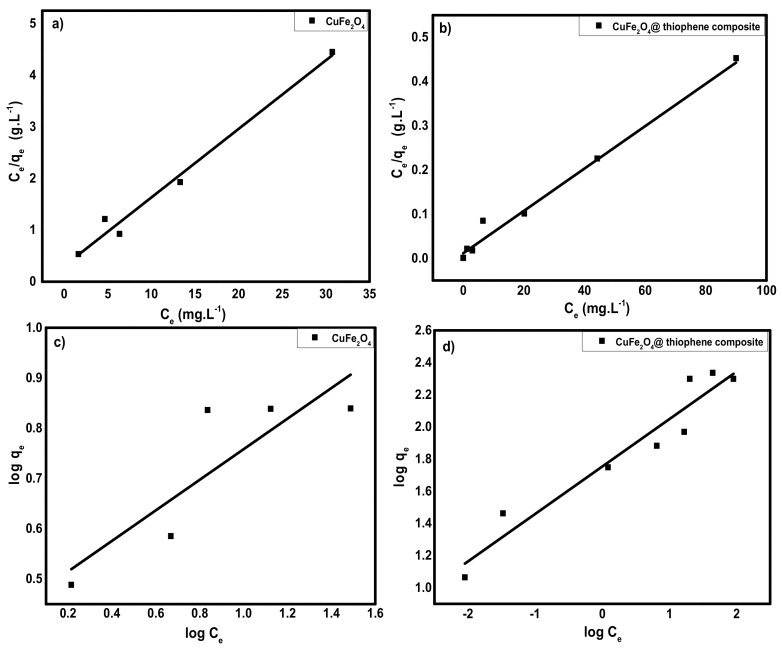
(**a**,**b**) Langmuir adsorption isotherm for adsorption of Hg(II) ions onto CuFe_2_O_4_ and CuFe_2_O_4_@Polythiophene composite; respectively and (**c**,**d**) Freundlich adsorption isotherm for adsorption of Hg(II) ions onto CuFe_2_O_4_ and CuFe_2_O_4_@Polythiophene composite; respectively.

As shown in Figure 12, Langmuir and Freundlich isotherms were used to designate the practical adsorption isotherm data. The isotherm parameters were illustrated in Table 3. It was observed that, both adsorbents obeyed Langmuir isotherms according to correlation coefficients (*R^2^*) of linear plot that confirmed the formation of monolayer on the surface the occurrence of homogeneous adsorption process of both adsorbents. From the Langmuir model, the maximum capacity per unit mass (*Q_m_*) was 53.7 and 208.77 mg/g for CuFe_2_O_4_ and CuFe_2_O_4_@Polythiophene composite, respectively.

#### 3.2.6. Reusability of CuFe_2_O_4_@Polythiophene Composite

**Figure 13:**
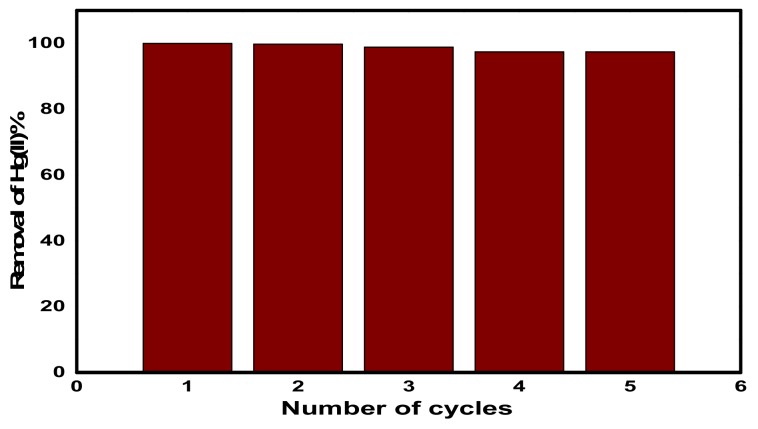
Hg(II) adsorption–desorption consecutive cycles on CuFe_2_O_4_@Polythiophene composite.

As shown in Figure 13**,** five adsorption–desorption cycles were carried out in order to examine the recyclability of CuFe_2_O_4_@Polythiophene composite and the removal percentage of Hg(II) was calculated by Equation (2). It was found that, there was not any obvious decrease in the removal efficiency after five cycles and the removal efficiency decreased gradually from 99.974% to 97.4% within consecutive cycles.

### 3.3. Adsorption Mechanism

**Figure 14:**
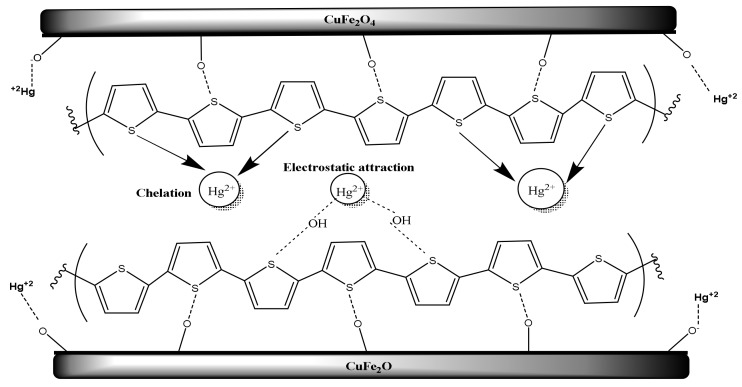
Schematic of Hg^2+^ adsorption mechanism on CuFe_2_O_4_@Polythiophene composite.

To illustrate the mechanism of Hg^2+^ removal by CuFe_2_O_4_@Polythiophene composite, Figure 14 is presented. There are two different suggested mechanisms are proposed here for the adsorption of Hg^2+^ on the surface of CuFe_2_O_4_@Polythiophene composite. The first is a physical adsorption on the surface of Polythiophene layer or in the porosity of the adsorbent. The last is a chemical adsorption through interactions of Polythiophene layer with Hg^2+^ ions. CuFe_2_O_4_ nanoparticles have a high specific surface area. Therefore, Hg^2+^ ions can penetrate through the adsorbent porosity and be adsorbed on the surface of CuFe_2_O_4_ nanoparticle. On the other hand, another probable adsorption mechanism occurring on the surface of Polythiophene is via chelation of Hg^2+^ ions with OH groups, bonded to sulfur on the structure of polythiophene [29,30,31].

## 4. Conclusions

For the first time, CuFe_2_O_4_@Polythiophene composite was synthesized and characterized. CuFe_2_O_4_@Polythiophene composite was compared with CuFe_2_O_4_ for the removal of Hg(II) from aqueous solution. It was observed that CuFe_2_O_4_@Polythiophene composite is more effective than CuFe_2_O_4_ in removal Hg(II) ions from aqueous solution. This may be attributed to the soft acid–soft base strong interaction between sulfur group in the polythiophene and Hg(II) ions. Also it was found that both adsorbents followed the second order model and Langmuir model with adsorption capacity of 7.53 and 208.77 mg/g for CuFe_2_O_4_ and CuFe_2_O_4_@Polythiophene composite, respectively. CuFe_2_O_4_@Polythiophene composite could be successfully regenerated after Hg(II) adsorption process with fast and simple manner and it could be used more than once and easily removed from aqueous solution by external magnetic field after adsorption process took place. CuFe_2_O_4_@Polythiophene composite is applicable for removal Hg(II) ions from aqueous solution.

## Figures and Tables

**Table 1:** Overall surface characteristic of proposed adsorbents that obtained by Brunauer–Emmett–Teller (BET) method.

Sample	* Surface Area (m^2^·g^−1^)	* Average Pore Volume (cm^3^·g^−1^)	* Average Pore Diameter (nm)
**CuFe_2_O_4_**	34.1 ± 2.3	0.10 ± 0.05	2.46 ± 0.3
**CuFe_2_O_4_@Polythiophene composite**	30.9 ± 1.5	0.06 ± 0.003	1.85 ± 0.2

* Average of 5 measurements.

**Table 2:** Adsorption kinetics parameters achieved using pseudo first order and second order models.

Adsorbent	Pseudo-First Order			Second Order			
	*k_1_* (min^−1^)	*q_e1_* (mg/g)	*R^2^*	*k_2_* (g/(mg·min))	*q_e2_* (mg/g)	*q_e_^exp^*	*R^2^*
**CuFe_2_O_4_**	0.00593	1.5896	0.91	5.12 × 10^−3^	6.0837	7.0452	0.993
**CuFe_2_O_4_@Polythiophene composite**	0.10287	3.4878	0.968	0.1273	9.9978	10.046	1

**Table 3:** Adsorption isotherm parameters achieved using Langmuir and Freundlich models.

Adsorbent	Langmuir Model			Freundlich Model		
	*Q_m_* (mg/g)	*b* (1/mg)	*R^2^* (*n =* 5)	*K_F_* (mg^(*n*−1)/*n*^L^1/*n*^g^−1^)	*n*	*R^2^* (*n* = 5)
**CuFe_2_O_4_**	7.5 ± 0.3	0.438	0.979	2.845	3.29	0.661
**CuFe_2_O_4_@Polythiophene composite**	208.7 ± 2.5	0.417	0.982	56.724	3.39	0.919
